# Dr. Watson on Cancer of the Stomach

**Published:** 1842-12-03

**Authors:** 


*Dr. Watson on Cancer of the Stomach.*—The case to which I allude was that of William Bedford, a man thirty-three years old, but looking much older, a farm labourer. He entered the hospital on the 30th of August, and many of the present auditory must remember him as a patient. At the period of his admission he was very thin and very feeble. His own statement, in reply to inquiries, was that he was affected with frequent vomiting—that he could retain nothing on his stomach—that no solid food could pass beyond a particular spot, which he pointed out at the lower part of the sternum—that the matter rejected at times appeared like phlegm, and subsequently had become a clear liquid like water—that some time antecedent to his admission he had been able occasionally to get a small morsel of solid food into the stomach—

that those occasions became gradually fewer, then altogether ceased—and that, for some time immediately prior to his coming to the hospital, he had been wholly unable to make the smallest morsel pass beyond the spot which he indicated—and that even the liquids to which he was compelled to resort for sustenance began to be rejected. At this time his bowels were sufficiently regular, his urine free, and not discoloured. It was very remarkable in this case, that the difficulty of retaining food on the stomach, or of even admitting it, had commenced at a very early age. The man was thirty-three years old when admitted to the hospital, and, from his statements, it had been ascertained that the difficulty had commenced full seven years before, when he was twenty-six years of age. The cancer, therefore—for such the disease was—had at that early period made such progress as to create the difficulty, which was experienced only occasionally at first; but, as the disease extended, the difficulty became more frequent, until finally ingestion of solid food into the stomach was utterly impossible, and every thing taken for sustenance was instantly rejected. When I examined the patient, on the day after his admission into the hospital, I found a sensible, defined hardness, across the epigastrium, which was very distinct on the left portion of that region. This resisting hard substance was somewhat tender when pressed upon. Slight pressure on the tumour occasionally caused a crepitating sound, produced by the displacement of flatus. This showed that it could not be formed by a solid mass—the liver, for example, or by the enlarged head of the pancreas, which I have known to occasion a prominent tumour in this situation. It was not an aneurism, for there was no pulsation, nor could we detect any trace of the whizzing sound which generally accompanies aneurism of the abdominal aorta. We concluded, in short, that the patient laboured under scirrhus of the stomach. Moreover, as the food never seemed to enter the stomach, and was rejected almost immediately on being taken, I set the case down as one of cancer affecting the cardiac orifice of that organ. When the pyloric orifice is implicated in any disease which diminishes the *outlet* of the stomach, or impedes the passage of food onwards into the duodenum, while the cardiac opening or *inlet* remains free, then the food enters the stomach readily enough, and is retained there for some time, undergoing digestion more or less perfectly; and it is after this—some time after a meal—when the digested aliment fails to find its way through the natural channel, that it is rejected by vomiting.

Nothing administered was found to do the man any good—that is, any sensible permanent good; nor, indeed, from the diagnosis was any expectation of good to be indulged in. On the 19th of September the patient complained of a good deal of pain just above the navel, and on pressing the finger suddenly downwards in this part of the abdomen, it came in contact with a hard and tender substance. Four days afterwards I could find no trace of hardness or tumour; the epigastrium, however, was more prominent, and was resonant on percussion; indeed, the abdomen was altogether fuller than it had been. On the 5th of October I again searched for the tumour and hardness, but in vain. By this time the man had become extremely emaciated; he was sinking rapidly, and on the 7th of October he died. As I before remarked to you, nothing made any favourable impression on him; he took the liquor potassæ at one time, then hydrocyanic acid, and, as he was unable to get down any solid nutriment I allowed him some wine and porter, and egg-flip; these he sipped by teaspoonfuls. I also endeavoured to support him by nutritive enemata, and for a few days immediately previous to his decease, enemata of beef-tea were thrown up; but this was found to cause so much distress and pain, without the possibility of effecting any good, that it was abandoned. For a few days before his death his appearance was remarkable; the face and neck were of a livid red, the tongue red and foul.

The diagnosis in this case was not very difficult, and was justified by what we found on examination after death. The cavity of the peritoneum contained a considerable quantity of a brownish coloured transparent fluid, of a very disagreeable odour. To the effusion of this liquid I attribute the fact that the tumour, which was partly formed by the stomach, and partly by omentum, ceased to be palpable at one time, though it was easily detected some time before. The lesson you may draw from this fact is, that negative signs, if I may so call them, are of less value than those which are positive. The absence of any apparent tumour was deceitful, but we were sure that it was there, because we had previously felt it. The tumour detected on the day of the patient's admission into hospital, and for three weeks subsequently, was formed by the indurated portion of the stomach; the second tumour, discovered on pressing the finger firmly above the umbilicus, was the omentum, which was rolled and matted together, and full of small masses of cancerous deposit. The stomach was small, and exhibited a fine specimen of scirrhus affecting both the cardiac and pyloric orifices of the organ. The lower part of the œsophagus and the upper portion of the duodenum were likewise involved in the disease. In addition to this, there was a growth of matter apparently cancerous, from the internal membrane of the stomach itself.

The mode of death in this case was particularly worthy of notice. It is not often that an opportunity occurs of observing a case of actual starvation, of death from total want of food, a gradual sinking under its effects. Such a phenomenon this case presented. During more than six weeks of his residence in this hospital the patient scarcely swallowed a morsel of solid food; he certainly retained none on the stomach; during the whole of the time he was kept alive by liquids, but he gradually wasted away; his heart acted with less and less vigour, and at length, while his intellectual faculties remained unimpaired, his pulse ceased to be felt at the wrists, and he expired from asthenia and syncope conjointly. One of the morbid symptoms of starvation is unusual fullness of the gall-bladder. That symptom was here developed; the fluid in the gall-bladder reached to the very edges of the orifice, and the bile was prevented from escaping, probably only by the pressure of the diseased and hardened parts on the excretory duct of the liver. Whether in analogous cases the bile is confined by some such mechanical cause or not, has not satisfactorily been ascertained. With all this quantity of bile, and the tendency to absorption that existed, there was no appearance of jaundice.

The case of William Bedford, then, was one of cancer; but on looking at the diseased parts now before you, you will see a false appearance of cancer, arising from hypertrophy of the muscular fibres which propel the digested food through the pyloric orifice of the stomach. Like all muscles impeded in action they have been excited to extraordinary efforts; and the increase of functional effort, according to a well-known law, leads to an increase of growth.

Cancer is a very obscure disease, and one of the most important subjects for the consideration of the pathologist. Its fatality and frequent occurrence, the acute pain by which it is generally attended, and its hereditary character, combine to render it of extreme interest. But another interest attaches to it from the obscure nature of the organic element of the disease, and from its peculiar mode of growth. Recently much light had been thrown on the subject, and it is to be hoped that still greater light is about to be thrown on it, for all pathologists looked with anxiety to the result of Mr. Kiernan's investigations, which I believe will shortly be placed before the profession. In the "Lancet," Dr. William Budd, of Bristol, has published two very interesting papers, to which I would direct your particular attention. They were published in two consecutive numbers of that journal for May last, and they are written, as is every thing from the pen of Dr. William Budd, extremely well. Whatever may be the causes of cancer, it appears to be clearly ascertained, that, however numerous or distant from each other the parts affected by it, the infection proceeds from one original tumour. It often occurs that the disease manifests itself in different parts of the body, sometimes contemporaneously, sometimes at different periods. But all these are but branches of the same malady. Generally, the internal parts are affected at a period subsequent to the appearance of the disease externally. Dr. B. has directed attention to the progress of cancer under some of its ordinary phases. Let us state an example: a small hard knot is detected in the female breast, lying loose in that organ; this tumour enlarges, grows, fastens on the parts around, no longer lies loose in the cellular tissue, but contracts adhesions with the surrounding parts, spreads out its claws, as it were, like a crab (hence the name cancer), seizes on the glands of the axilla, and disseminates small tumours through the viscera. On examination after death, cancerous matter is detected in the viscera and lymphatic glands, and is frequently found in the lungs, the liver, and peritoneum. From these facts, and from the circumstance that the veins are found charged with the same cancerous matter, Dr. Budd infers (and a most important inference it is, if drawn from a sufficient number of authenticated facts) that the secondary tumours discovered in the viscera, and other parts so diseased, are derived from the first tumour—are caused by seeds from the primary or parent growth, conveyed by the blood to those localities, and form themselves new centres for the further dissemination of the complaint. Some curious results of the microscopic investigation of cancerous matter are adduced in aid of the hypotheses. The labours of Müller, who has investigated the minute anatomy of cancer by the help of the microscope, show that the substance of cancerous tumour is a soft kind of pulp, held loosely together by a fibrous web. The pulpy matter presents an organised form of an extremely interesting kind, being found to be almost entirely composed of minute globular cells, containing within their cavities a vast number of very minute granules.

Similar cells are found in the organization of portions of the vegetable kingdom. Those granules are supposed to be what are called cysto-blasts, or germs of new cells, and becoming detached from their parent cells, are carried about until deposited in some place where, from their size, they are unable to pass through the smaller capillary vessels, and there they become parent cells, and engender new growths. This is a consideration of immense practical importance, as, if fully established, it would of itself demonstrate the necessity of immediate removal of the first cancerous tumour by the knife, and of its complete removal. The timely extirpation of cancerous growths is thus indispensable. In very many cases removal by the knife has proved fully successful in wholly eradicating the disease; in many cases it has not been successful. But in the latter it is inferred that the removal of the cancerous matter had not been complete—that either the operation had been deferred until the germs had been disseminated, or thatt the knife had not removed the whole of the diseased matter. So very minute are those germs, that it is altogether impossible to detect them, unless by means of miscropic power; and Dr. Budd recommends that the surface should be subjected to microscopic examination, as if on that surface be found any of those cells, the clear inference is, that others have been left behind, and consequently that the removal has been incomplete. In those operations the surgeon should cut away not only enough, but what may be called more than enough.

This subject, gentlemen, is altogether most interesting; but it requires further examination. What the exact nature, and what the origin of those minute cells, are as yet uncertain; they may be termed a sort of hydatids. They appear, like plants, gifted with an independent vitality, possessing the power of generation, increasing and multiplying prodigiously, and capable of being conveyed to remote distances from the parent growth, without losing their character or powers; they might indeed be appropriately called a sort of parasitic animal. What is the origin of the primary or parent growth, it is, at least as yet, impossible to say. Some authors contended that cancer is infectious and contagious. Dr. Budd states that Langenbeck injected cancerous pulp from a living body into the veins of a dog; the animal after some time wasted away and died, and on examination several cancerous tumors were found in his lungs. Soot is supposed to be a cause of cancer, and one species of that disease is known as chimney-sweeper's cancer. Dr. Budd has alluded to cases of cancer of the penis in men, whose wives laboured under cancer of the uterus, which would tend to establish the doctrine of its being contagious. The germs of cancer clearly possess an independent vitality, and are like parasitic animals, or a kind of fungous growth, perhaps somewhat resembling the disease known as scald head, which is now held to depend on a sort of crytogamic plant, the *habitat* or one of the *habitats* of which is the human head. As I totally disbelieve the doctrine of spontaneous generation, I cannot yield to the suggestion that these germs originate in the body, but am of opinion that they are introduced by some yet unascertained mode. The introduction of insects into the human frame by unknown means is familiar to us all, and from all that is known I am induced to believe that cancerous matter is similarly introduced. In conclusion, I would again impress on the student the necessity of pursuing this important investigation, and again strongly recommend to him a careful perusal of the whole essay of Dr. Budd, as most interesting and instructive.—*Prov. Med. Journ.*, Oct. 29, 1842.

